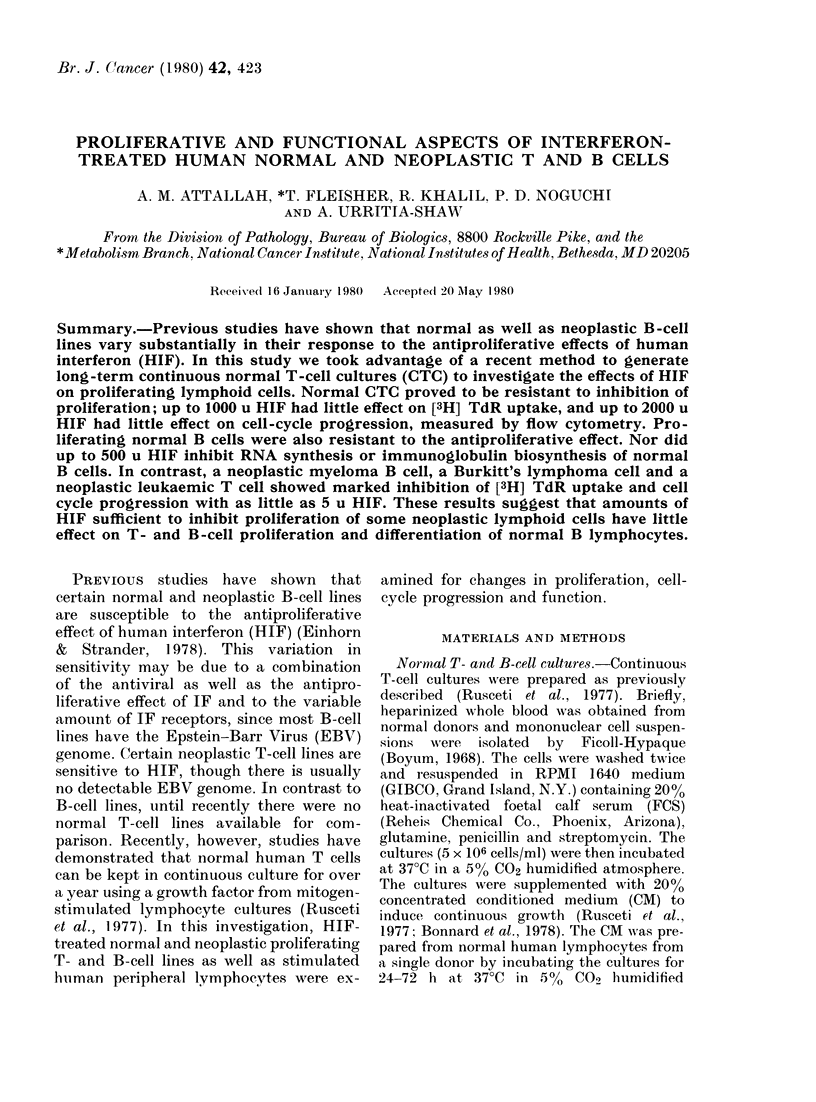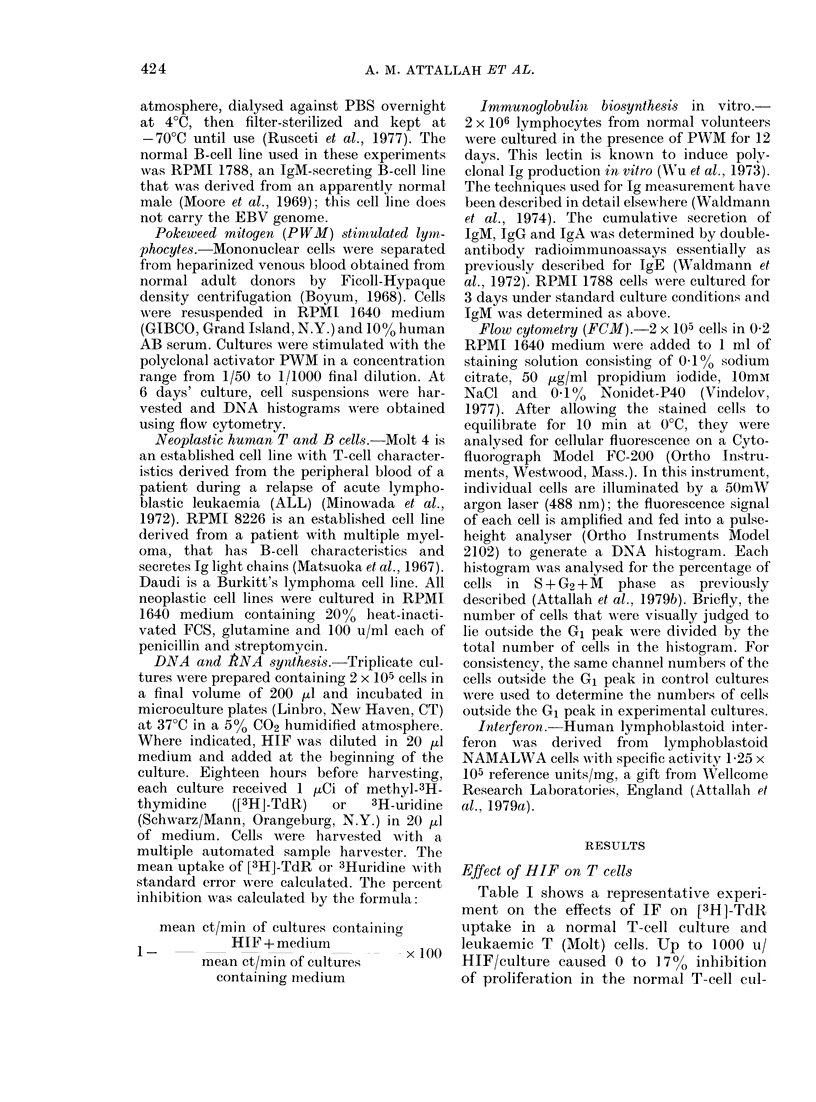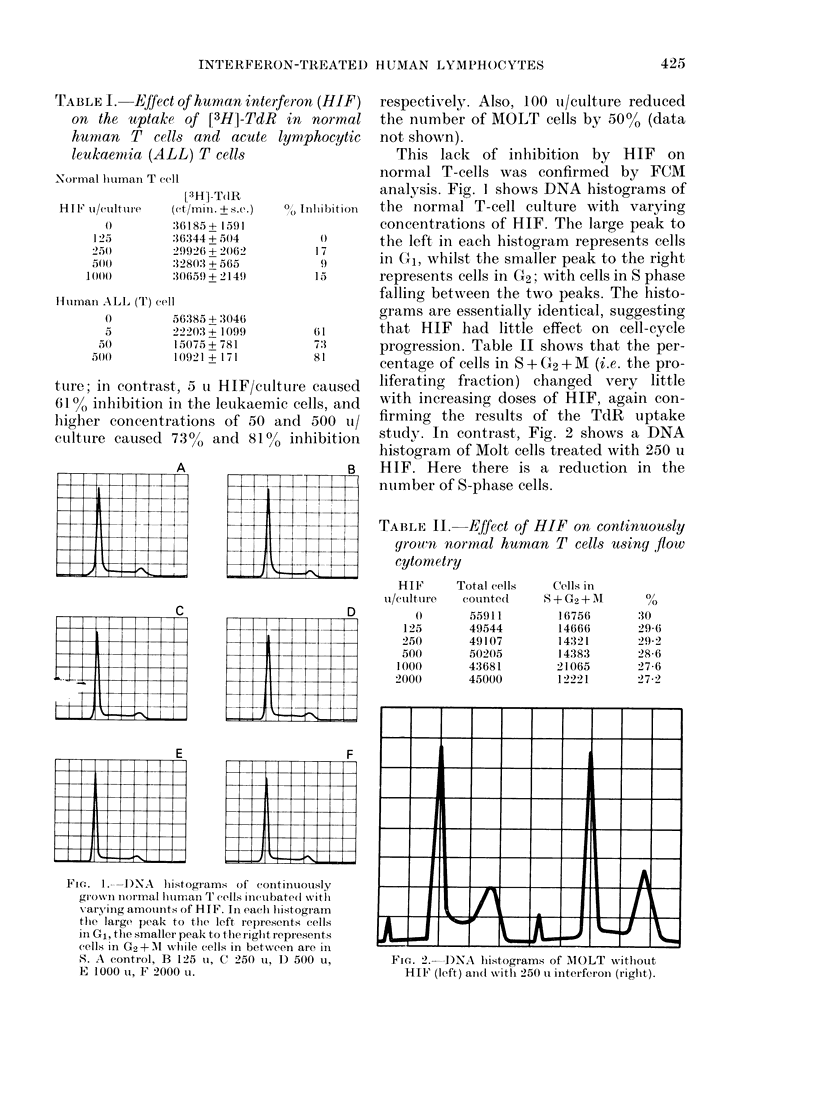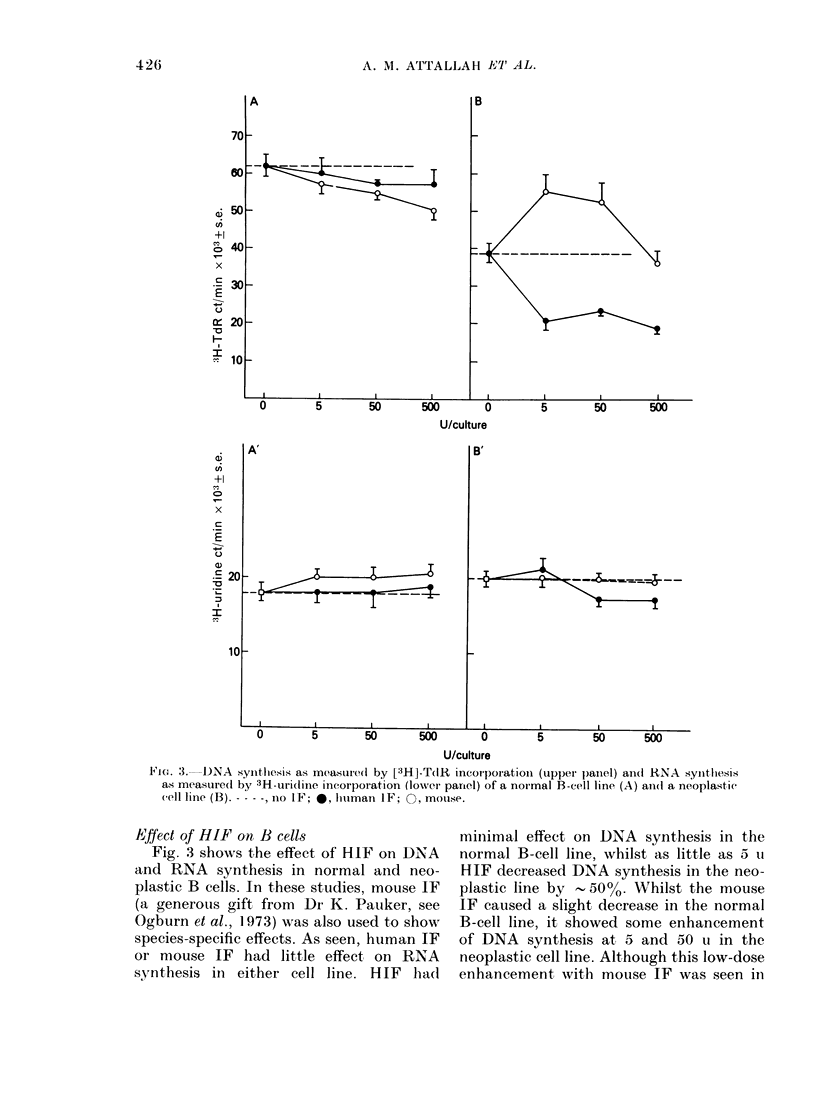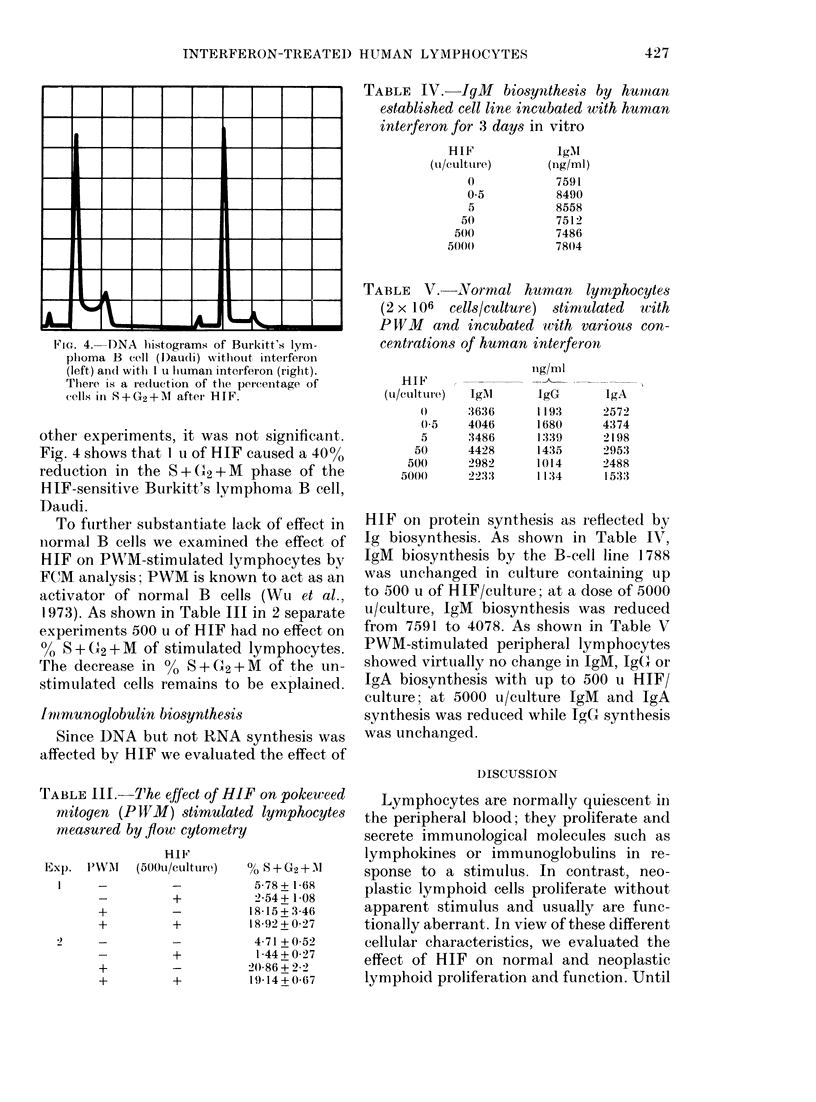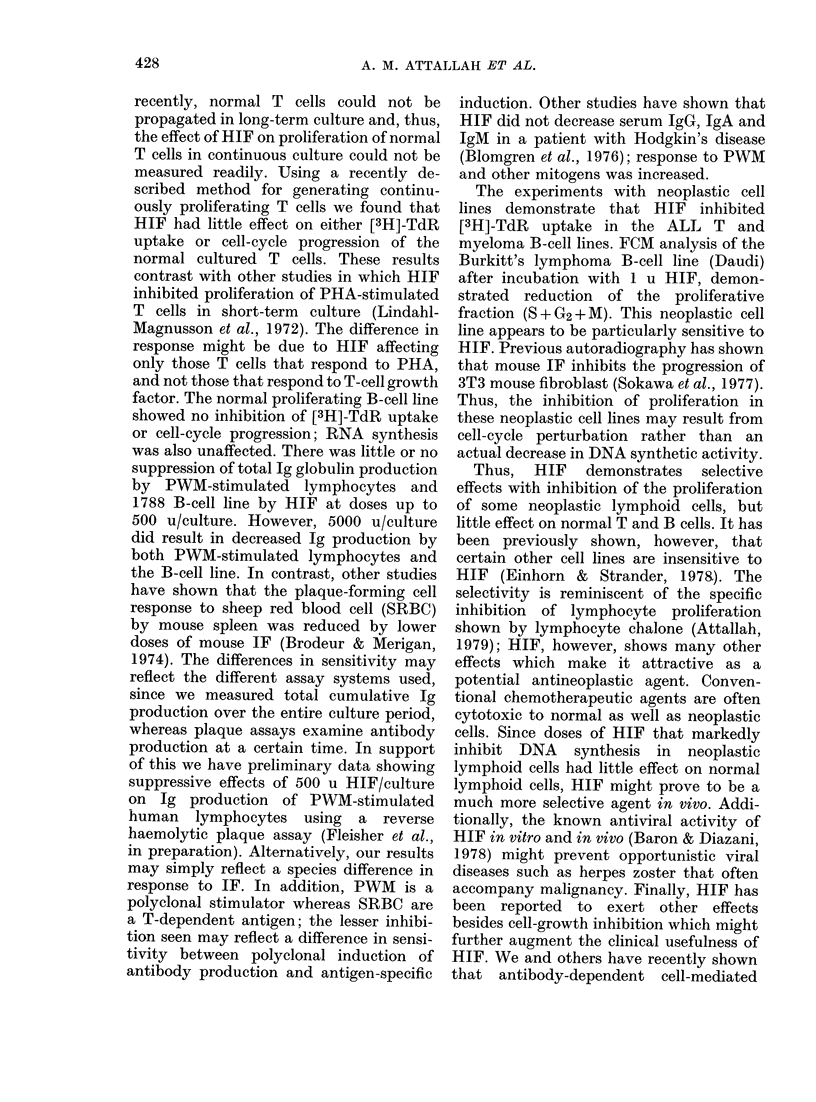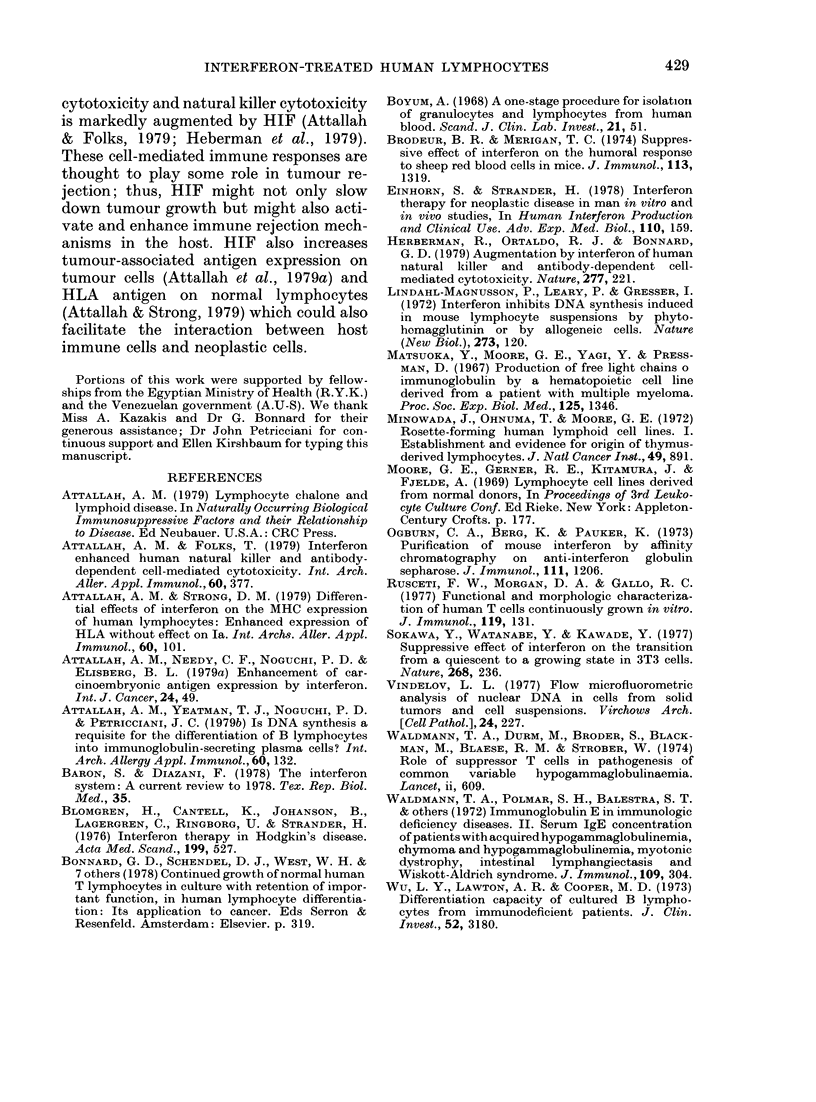# Proliferative and functional aspects of interferon-treated human normal and neoplastic T and B cells.

**DOI:** 10.1038/bjc.1980.254

**Published:** 1980-09

**Authors:** A. M. Attallah, T. Fleisher, R. Khalil, P. D. Noguchi, A. Urritia-Shaw

## Abstract

Previous studies have shown that normal as well as neoplastic B-cell lines vary substantially in their response to the antiproliferative effects of human interferon (HIF). In this study we took advantage of a recent method to generate long-term continuous normal T-cell cultures (CTC) to investigate the effects of HIF on proliferating lymphoid cells. Normal CTC proved to be resistant to inhibition of proliferation; up to 1000 u HIF had little effect on [3H] TdR uptake, and up to 2000 u HIF had little effect on cell-cycle progression, measured by flow cytometry. Proliferating normal B cells were also resistant to the antiproliferative effect. Nor did up to 500 m HIF inhibit RNA synthesis or immunoglobulin biosynthesis of normal B cells. In contrast, a neoplastic myeloma B cell, a Burkitt's lymphoma cell and a neoplastic leukaemic T cell showed marked inhibition of [3H] TdR uptake and cell cycle progression with as little as 5 u HIF. These results suggest that amounts of HIF sufficient to inhibit proliferation of some neoplastic lymphoid cells have little effect on T- and B-cell proliferation and differentiation of normal B lymphocytes.


Br. J. Cancer (1980) 42, 423

### PROLIFERATIVE AND FUNCTIONAL ASPECTS OF INTERFERON-TREATED HUMAN NORMAL AND NEOPLASTIC T AND B CELLS

A. M. ATTALLAH, *T. FLEISHER, R. KHALIL, P. D. NOGUCHI AND A. URRITIA-SHAW

*From the Division of Pathology, Bureau of Biologics, 8800 Rockville Pike, and the *Metabolism Branch, National Cancer Institute, National Institutes of Health, Bethesda, MD 20205*

Received 16 January 1980 Accepted 20 May 1980

**Summary.—Previous studies have shown that normal as well as neoplastic B-cell lines vary substantially in their response to the antiproliferative effects of human interferon (HIF). In this study we took advantage of a recent method to generate long-term continuous normal T-cell cultures (CTC) to investigate the effects of HIF on proliferating lymphoid cells. Normal CTC proved to be resistant to inhibition of proliferation; up to 1000 u HIF had little effect on [$^3$H] TdR uptake, and up to 2000 u HIF had little effect on cell-cycle progression, measured by flow cytometry. Proliferating normal B cells were also resistant to the antiproliferative effect. Nor did up to 500 u HIF inhibit RNA synthesis or immunoglobulin biosynthesis of normal B cells. In contrast, a neoplastic myeloma B cell, a Burkitt's lymphoma cell and a neoplastic leukaemic T cell showed marked inhibition of [$^3$H] TdR uptake and cell cycle progression with as little as 5 u HIF. These results suggest that amounts of HIF sufficient to inhibit proliferation of some neoplastic lymphoid cells have little effect on T- and B-cell proliferation and differentiation of normal B lymphocytes.**

PREVIOUS studies have shown that certain normal and neoplastic B-cell lines are susceptible to the antiproliferative effect of human interferon (HIF) (Einhorn & Strander, 1978). This variation in sensitivity may be due to a combination of the antiviral as well as the antiproliferative effect of IF and to the variable amount of IF receptors, since most B-cell lines have the Epstein–Barr Virus (EBV) genome. Certain neoplastic T-cell lines are sensitive to HIF, though there is usually no detectable EBV genome. In contrast to B-cell lines, until recently there were no normal T-cell lines available for comparison. Recently, however, studies have demonstrated that normal human T cells can be kept in continuous culture for over a year using a growth factor from mitogen-stimulated lymphocyte cultures (Rusceti *et al.*, 1977). In this investigation, HIF-treated normal and neoplastic proliferating T- and B-cell lines as well as stimulated human peripheral lymphocytes were examined for changes in proliferation, cell-cycle progression and function.

### MATERIALS AND METHODS

*Normal T- and B-cell cultures.*—Continuous T-cell cultures were prepared as previously described (Rusceti *et al.*, 1977). Briefly, heparinized whole blood was obtained from normal donors and mononuclear cell suspensions were isolated by Ficoll-Hypaque (Boyum, 1968). The cells were washed twice and resuspended in RPMI 1640 medium (GIBCO, Grand Island, N.Y.) containing 20% heat-inactivated foetal calf serum (FCS) (Reheis Chemical Co., Phoenix, Arizona), glutamine, penicillin and streptomycin. The cultures ($5 \times 10^6$ cells/ml) were then incubated at 37°C in a 5% $CO_2$ humidified atmosphere. The cultures were supplemented with 20% concentrated conditioned medium (CM) to induce continuous growth (Rusceti *et al.*, 1977; Bonnard *et al.*, 1978). The CM was prepared from normal human lymphocytes from a single donor by incubating the cultures for 24–72 h at 37°C in 5% $CO_2$ humidified atmosphere, dialysed against PBS overnight at 4°C, then filter-sterilized and kept at −70°C until use (Rusceti *et al.*, 1977). The normal B-cell line used in these experiments was RPMI 1788, an IgM-secreting B-cell line that was derived from an apparently normal male (Moore *et al.*, 1969); this cell line does not carry the EBV genome.

*Pokeweed mitogen (PWM) stimulated lymphocytes.*—Mononuclear cells were separated from heparinized venous blood obtained from normal adult donors by Ficoll-Hypaque density centrifugation (Boyum, 1968). Cells were resuspended in RPMI 1640 medium (GIBCO, Grand Island, N.Y.) and 10% human AB serum. Cultures were stimulated with the polyclonal activator PWM in a concentration range from 1/50 to 1/1000 final dilution. At 6 days' culture, cell suspensions were harvested and DNA histograms were obtained using flow cytometry.

*Neoplastic human T and B cells.*—Molt 4 is an established cell line with T-cell characteristics derived from the peripheral blood of a patient during a relapse of acute lymphoblastic leukaemia (ALL) (Minowada *et al.*, 1972). RPMI 8226 is an established cell line derived from a patient with multiple myeloma, that has B-cell characteristics and secretes Ig light chains (Matsuoka *et al.*, 1967). Daudi is a Burkitt's lymphoma cell line. All neoplastic cell lines were cultured in RPMI 1640 medium containing 20% heat-inactivated FCS, glutamine and 100 u/ml each of penicillin and streptomycin.

*DNA and RNA synthesis.*—Triplicate cultures were prepared containing $2 \times 10^5$ cells in a final volume of 200 $\mu$l and incubated in microculture plates (Linbro, New Haven, CT) at 37°C in a 5% $CO_2$ humidified atmosphere. Where indicated, HIF was diluted in 20 $\mu$l medium and added at the beginning of the culture. Eighteen hours before harvesting, each culture received 1 $\mu$Ci of methyl-$^3$H-thymidine ([$^3$H]-TdR) or $^3$H-uridine (Schwarz/Mann, Orangeburg, N.Y.) in 20 $\mu$l of medium. Cells were harvested with a multiple automated sample harvester. The mean uptake of [$^3$H]-TdR or $^3$Huridine with standard error were calculated. The percent inhibition was calculated by the formula:

$$1 - \frac{\text{mean ct/min of cultures containing HIF+medium}}{\text{mean ct/min of cultures containing medium}} \times 100$$

*Immunoglobulin biosynthesis* in vitro.—$2 \times 10^6$ lymphocytes from normal volunteers were cultured in the presence of PWM for 12 days. This lectin is known to induce polyclonal Ig production *in vitro* (Wu *et al.*, 1973). The techniques used for Ig measurement have been described in detail elsewhere (Waldmann *et al.*, 1974). The cumulative secretion of IgM, IgG and IgA was determined by double-antibody radioimmunoassays essentially as previously described for IgE (Waldmann *et al.*, 1972). RPMI 1788 cells were cultured for 3 days under standard culture conditions and IgM was determined as above.

*Flow cytometry (FCM).*—$2 \times 10^5$ cells in 0·2 RPMI 1640 medium were added to 1 ml of staining solution consisting of 0·1% sodium citrate, 50 $\mu$g/ml propidium iodide, 10mM NaCl and 0·1% Nonidet-P40 (Vindelov, 1977). After allowing the stained cells to equilibrate for 10 min at 0°C, they were analysed for cellular fluorescence on a Cytofluorograph Model FC-200 (Ortho Instruments, Westwood, Mass.). In this instrument, individual cells are illuminated by a 50mW argon laser (488 nm); the fluorescence signal of each cell is amplified and fed into a pulse-height analyser (Ortho Instruments Model 2102) to generate a DNA histogram. Each histogram was analysed for the percentage of cells in $S+G_2+M$ phase as previously described (Attallah *et al.*, 1979*b*). Briefly, the number of cells that were visually judged to lie outside the $G_1$ peak were divided by the total number of cells in the histogram. For consistency, the same channel numbers of the cells outside the $G_1$ peak in control cultures were used to determine the numbers of cells outside the $G_1$ peak in experimental cultures.

*Interferon.*—Human lymphoblastoid interferon was derived from lymphoblastoid NAMALWA cells with specific activity $1{\cdot}25 \times 10^5$ reference units/mg, a gift from Wellcome Research Laboratories, England (Attallah *et al.*, 1979*a*).

### RESULTS

### *Effect of HIF on T cells*

Table I shows a representative experiment on the effects of IF on [$^3$H]-TdR uptake in a normal T-cell culture and leukaemic T (Molt) cells. Up to 1000 u/ HIF/culture caused 0 to 17% inhibition of proliferation in the normal T-cell cul-

TABLE I.—*Effect of human interferon (HIF) on the uptake of [$^3$H]-TdR in normal human T cells and acute lymphocytic leukaemia (ALL) T cells*

| HIF u/culture | [$^3$H]-TdR (ct/min. ± s.e.) | % Inhibition |
|---|---|---|
| Normal human T cell | | |
| 0 | 36185 ± 1591 | |
| 125 | 36344 ± 504 | 0 |
| 250 | 29926 ± 2062 | 17 |
| 500 | 32803 ± 565 | 9 |
| 1000 | 30659 ± 2149 | 15 |
| Human ALL (T) cell | | |
| 0 | 56385 ± 3046 | |
| 5 | 22203 ± 1099 | 61 |
| 50 | 15075 ± 781 | 73 |
| 500 | 10921 ± 171 | 81 |

ture; in contrast, 5 u HIF/culture caused 61% inhibition in the leukaemic cells, and higher concentrations of 50 and 500 u/culture caused 73% and 81% inhibition

FIG. 1.—DNA histograms of continuously grown normal human T cells incubated with varying amounts of HIF. In each histogram the large peak to the left represents cells in $G_1$, the smaller peak to the right represents cells in $G_2$+M while cells in between are in S. A control, B 125 u, C 250 u, D 500 u, E 1000 u, F 2000 u.

respectively. Also, 100 u/culture reduced the number of MOLT cells by 50% (data not shown).

This lack of inhibition by HIF on normal T-cells was confirmed by FCM analysis. Fig. 1 shows DNA histograms of the normal T-cell culture with varying concentrations of HIF. The large peak to the left in each histogram represents cells in $G_1$, whilst the smaller peak to the right represents cells in $G_2$; with cells in S phase falling between the two peaks. The histograms are essentially identical, suggesting that HIF had little effect on cell-cycle progression. Table II shows that the percentage of cells in S + $G_2$ + M (*i.e.* the proliferating fraction) changed very little with increasing doses of HIF, again confirming the results of the TdR uptake study. In contrast, Fig. 2 shows a DNA histogram of Molt cells treated with 250 u HIF. Here there is a reduction in the number of S-phase cells.

TABLE II.—*Effect of HIF on continuously grown normal human T cells using flow cytometry*

| HIF u/culture | Total cells counted | Cells in S + $G_2$ + M | % |
|---|---|---|---|
| 0 | 55911 | 16756 | 30 |
| 125 | 49544 | 14666 | 29·6 |
| 250 | 49107 | 14321 | 29·2 |
| 500 | 50205 | 14383 | 28·6 |
| 1000 | 43681 | 21065 | 27·6 |
| 2000 | 45000 | 12221 | 27·2 |

FIG. 2.—DNA histograms of MOLT without HIF (left) and with 250 u interferon (right).

FIG. 3.—DNA synthesis as measured by [$^3$H]-TdR incorporation (upper panel) and RNA synthesis as measured by $^3$H-uridine incorporation (lower panel) of a normal B-cell line (A) and a neoplastic cell line (B). - - - -, no IF; ●, human IF; ○, mouse.

*Effect of HIF on B cells*

Fig. 3 shows the effect of HIF on DNA and RNA synthesis in normal and neoplastic B cells. In these studies, mouse IF (a generous gift from Dr K. Pauker, see Ogburn *et al.*, 1973) was also used to show species-specific effects. As seen, human IF or mouse IF had little effect on RNA synthesis in either cell line. HIF had minimal effect on DNA synthesis in the normal B-cell line, whilst as little as 5 u HIF decreased DNA synthesis in the neoplastic line by $\sim 50\%$. Whilst the mouse IF caused a slight decrease in the normal B-cell line, it showed some enhancement of DNA synthesis at 5 and 50 u in the neoplastic cell line. Although this low-dose enhancement with mouse IF was seen in

FIG. 4.—DNA histograms of Burkitt's lymphoma B cell (Daudi) without interferon (left) and with 1 u human interferon (right). There is a reduction of the percentage of cells in $S+G_2+M$ after HIF.

other experiments, it was not significant. Fig. 4 shows that 1 u of HIF caused a 40% reduction in the $S+G_2+M$ phase of the HIF-sensitive Burkitt's lymphoma B cell, Daudi.

To further substantiate lack of effect in normal B cells we examined the effect of HIF on PWM-stimulated lymphocytes by FCM analysis; PWM is known to act as an activator of normal B cells (Wu *et al.*, 1973). As shown in Table III in 2 separate experiments 500 u of HIF had no effect on % $S+G_2+M$ of stimulated lymphocytes. The decrease in % $S+G_2+M$ of the unstimulated cells remains to be explained.

*Immunoglobulin biosynthesis*

Since DNA but not RNA synthesis was affected by HIF we evaluated the effect of HIF on protein synthesis as reflected by Ig biosynthesis. As shown in Table IV, IgM biosynthesis by the B-cell line 1788 was unchanged in culture containing up to 500 u of HIF/culture; at a dose of 5000 u/culture, IgM biosynthesis was reduced from 7591 to 4078. As shown in Table V PWM-stimulated peripheral lymphocytes showed virtually no change in IgM, IgG or IgA biosynthesis with up to 500 u HIF/culture; at 5000 u/culture IgM and IgA synthesis was reduced while IgG synthesis was unchanged.

TABLE III.—*The effect of HIF on pokeweed mitogen (PWM) stimulated lymphocytes measured by flow cytometry*

| Exp. | PWM | HIF (500u/culture) | % $S+G_2+M$ |
|---|---|---|---|
| 1 | − | − | 5·78 ± 1·68 |
| | − | + | 2·54 ± 1·08 |
| | + | − | 18·15 ± 3·46 |
| | + | + | 18·92 ± 0·27 |
| 2 | − | − | 4·71 ± 0·52 |
| | − | + | 1·44 ± 0·27 |
| | + | − | 20·86 ± 2·2 |
| | + | + | 19·14 ± 0·67 |

TABLE IV.—*IgM biosynthesis by human established cell line incubated with human interferon for 3 days* in vitro

| HIF (u/culture) | IgM (ng/ml) |
|---|---|
| 0 | 7591 |
| 0·5 | 8490 |
| 5 | 8558 |
| 50 | 7512 |
| 500 | 7486 |
| 5000 | 7804 |

TABLE V.—*Normal human lymphocytes* ($2\times10^6$ *cells/culture) stimulated with PWM and incubated with various concentrations of human interferon*

| HIF | ng/ml | | |
|---|---|---|---|
| (u/culture) | IgM | IgG | IgA |
| 0 | 3636 | 1193 | 2572 |
| 0·5 | 4046 | 1680 | 4374 |
| 5 | 3486 | 1339 | 2198 |
| 50 | 4428 | 1435 | 2953 |
| 500 | 2982 | 1014 | 2488 |
| 5000 | 2233 | 1134 | 1533 |

### DISCUSSION

Lymphocytes are normally quiescent in the peripheral blood; they proliferate and secrete immunological molecules such as lymphokines or immunoglobulins in response to a stimulus. In contrast, neoplastic lymphoid cells proliferate without apparent stimulus and usually are functionally aberrant. In view of these different cellular characteristics, we evaluated the effect of HIF on normal and neoplastic lymphoid proliferation and function. Until recently, normal T cells could not be propagated in long-term culture and, thus, the effect of HIF on proliferation of normal T cells in continuous culture could not be measured readily. Using a recently described method for generating continuously proliferating T cells we found that HIF had little effect on either [$^3$H]-TdR uptake or cell-cycle progression of the normal cultured T cells. These results contrast with other studies in which HIF inhibited proliferation of PHA-stimulated T cells in short-term culture (Lindahl-Magnusson *et al.*, 1972). The difference in response might be due to HIF affecting only those T cells that respond to PHA, and not those that respond to T-cell growth factor. The normal proliferating B-cell line showed no inhibition of [$^3$H]-TdR uptake or cell-cycle progression; RNA synthesis was also unaffected. There was little or no suppression of total Ig globulin production by PWM-stimulated lymphocytes and 1788 B-cell line by HIF at doses up to 500 u/culture. However, 5000 u/culture did result in decreased Ig production by both PWM-stimulated lymphocytes and the B-cell line. In contrast, other studies have shown that the plaque-forming cell response to sheep red blood cell (SRBC) by mouse spleen was reduced by lower doses of mouse IF (Brodeur & Merigan, 1974). The differences in sensitivity may reflect the different assay systems used, since we measured total cumulative Ig production over the entire culture period, whereas plaque assays examine antibody production at a certain time. In support of this we have preliminary data showing suppressive effects of 500 u HIF/culture on Ig production of PWM-stimulated human lymphocytes using a reverse haemolytic plaque assay (Fleisher *et al.*, in preparation). Alternatively, our results may simply reflect a species difference in response to IF. In addition, PWM is a polyclonal stimulator whereas SRBC are a T-dependent antigen; the lesser inhibition seen may reflect a difference in sensitivity between polyclonal induction of antibody production and antigen-specific induction. Other studies have shown that HIF did not decrease serum IgG, IgA and IgM in a patient with Hodgkin's disease (Blomgren *et al.*, 1976); response to PWM and other mitogens was increased.

The experiments with neoplastic cell lines demonstrate that HIF inhibited [$^3$H]-TdR uptake in the ALL T and myeloma B-cell lines. FCM analysis of the Burkitt's lymphoma B-cell line (Daudi) after incubation with 1 u HIF, demonstrated reduction of the proliferative fraction ($S+G_2+M$). This neoplastic cell line appears to be particularly sensitive to HIF. Previous autoradiography has shown that mouse IF inhibits the progression of 3T3 mouse fibroblast (Sokawa *et al.*, 1977). Thus, the inhibition of proliferation in these neoplastic cell lines may result from cell-cycle perturbation rather than an actual decrease in DNA synthetic activity.

Thus, HIF demonstrates selective effects with inhibition of the proliferation of some neoplastic lymphoid cells, but little effect on normal T and B cells. It has been previously shown, however, that certain other cell lines are insensitive to HIF (Einhorn & Strander, 1978). The selectivity is reminiscent of the specific inhibition of lymphocyte proliferation shown by lymphocyte chalone (Attallah, 1979); HIF, however, shows many other effects which make it attractive as a potential antineoplastic agent. Conventional chemotherapeutic agents are often cytotoxic to normal as well as neoplastic cells. Since doses of HIF that markedly inhibit DNA synthesis in neoplastic lymphoid cells had little effect on normal lymphoid cells, HIF might prove to be a much more selective agent *in vivo*. Additionally, the known antiviral activity of HIF *in vitro* and *in vivo* (Baron & Diazani, 1978) might prevent opportunistic viral diseases such as herpes zoster that often accompany malignancy. Finally, HIF has been reported to exert other effects besides cell-growth inhibition which might further augment the clinical usefulness of HIF. We and others have recently shown that antibody-dependent cell-mediated cytotoxicity and natural killer cytotoxicity is markedly augmented by HIF (Attallah & Folks, 1979; Heberman *et al.*, 1979). These cell-mediated immune responses are thought to play some role in tumour rejection; thus, HIF might not only slow down tumour growth but might also activate and enhance immune rejection mechanisms in the host. HIF also increases tumour-associated antigen expression on tumour cells (Attallah *et al.*, 1979*a*) and HLA antigen on normal lymphocytes (Attallah & Strong, 1979) which could also facilitate the interaction between host immune cells and neoplastic cells.

Portions of this work were supported by fellowships from the Egyptian Ministry of Health (R.Y.K.) and the Venezuelan government (A.U-S). We thank Miss A. Kazakis and Dr G. Bonnard for their generous assistance; Dr John Petricciani for continuous support and Ellen Kirshbaum for typing this manuscript.

REFERENCES

ATTALLAH, A. M. (1979) Lymphocyte chalone and lymphoid disease. In *Naturally Occurring Biological Immunosuppressive Factors and their Relationship to Disease.* Ed Neubauer. U.S.A.: CRC Press.

ATTALLAH, A. M. & FOLKS, T. (1979) Interferon enhanced human natural killer and antibody-dependent cell-mediated cytotoxicity. *Int. Arch. Aller. Appl. Immunol.*, **60**, 377.

ATTALLAH, A. M. & STRONG, D. M. (1979) Differential effects of interferon on the MHC expression of human lymphocytes: Enhanced expression of HLA without effect on Ia. *Int. Archs. Aller. Appl. Immunol.*, **60**, 101.

ATTALLAH, A. M., NEEDY, C. F., NOGUCHI, P. D. & ELISBERG, B. L. (1979*a*) Enhancement of carcinoembryonic antigen expression by interferon. *Int. J. Cancer*, **24**, 49.

ATTALLAH, A. M., YEATMAN, T. J., NOGUCHI, P. D. & PETRICCIANI, J. C. (1979*b*) Is DNA synthesis a requisite for the differentiation of B lymphocytes into immunoglobulin-secreting plasma cells? *Int. Arch. Allergy Appl. Immunol.*, **60**, 132.

BARON, S. & DIAZANI, F. (1978) The interferon system: A current review to 1978. *Tex. Rep. Biol. Med.*, **35**.

BLOMGREN, H., CANTELL, K., JOHANSON, B., LAGERGREN, C., RINGBORG, U. & STRANDER, H. (1976) Interferon therapy in Hodgkin's disease. *Acta Med. Scand.*, **199**, 527.

BONNARD, G. D., SCHENDEL, D. J., WEST, W. H. & 7 others (1978) Continued growth of normal human T lymphocytes in culture with retention of important function, in human lymphocyte differentiation: Its application to cancer. Eds Serron & Resenfeld. Amsterdam: Elsevier. p. 319.

BOYUM, A. (1968) A one-stage procedure for isolation of granulocytes and lymphocytes from human blood. *Scand. J. Clin. Lab. Invest.*, **21**, 51.

BRODEUR, B. R. & MERIGAN, T. C. (1974) Suppressive effect of interferon on the humoral response to sheep red blood cells in mice. *J. Immunol.*, **113**, 1319.

EINHORN, S. & STRANDER, H. (1978) Interferon therapy for neoplastic disease in man *in vitro* and *in vivo* studies, In *Human Interferon Production and Clinical Use. Adv. Exp. Med. Biol.*, **110**, 159.

HERBERMAN, R., ORTALDO, R. J. & BONNARD, G. D. (1979) Augmentation by interferon of human natural killer and antibody-dependent cell-mediated cytotoxicity. *Nature*, **277**, 221.

LINDAHL-MAGNUSSON, P., LEARY, P. & GRESSER, I. (1972) Interferon inhibits DNA synthesis induced in mouse lymphocyte suspensions by phytohemagglutinin or by allogeneic cells. *Nature (New Biol.)*, **273**, 120.

MATSUOKA, Y., MOORE, G. E., YAGI, Y. & PRESSMAN, D. (1967) Production of free light chains o immunoglobulin by a hematopoietic cell line derived from a patient with multiple myeloma. *Proc. Soc. Exp. Biol. Med.*, **125**, 1346.

MINOWADA, J., OHNUMA, T. & MOORE, G. E. (1972) Rosette-forming human lymphoid cell lines. I. Establishment and evidence for origin of thymus-derived lymphocytes. *J. Natl Cancer Inst.*, **49**, 891.

MOORE, G. E., GERNER, R. E., KITAMURA, J. & FJELDE, A. (1969) Lymphocyte cell lines derived from normal donors, In *Proceedings of 3rd Leukocyte Culture Conf.* Ed Rieke. New York: Appleton-Century Crofts. p. 177.

OGBURN, C. A., BERG, K. & PAUKER, K. (1973) Purification of mouse interferon by affinity chromatography on anti-interferon globulin sepharose. *J. Immunol.*, **111**, 1206.

RUSCETI, F. W., MORGAN, D. A. & GALLO, R. C. (1977) Functional and morphologic characterization of human T cells continuously grown *in vitro*. *J. Immunol.*, **119**, 131.

SOKAWA, Y., WATANABE, Y. & KAWADE, Y. (1977) Suppressive effect of interferon on the transition from a quiescent to a growing state in 3T3 cells. *Nature*, **268**, 236.

VINDELOV, L. L. (1977) Flow microfluorometric analysis of nuclear DNA in cells from solid tumors and cell suspensions. *Virchows Arch. [Cell Pathol.]*, **24**, 227.

WALDMANN, T. A., DURM, M., BRODER, S., BLACKMAN, M., BLAESE, R. M. & STROBER, W. (1974) Role of suppressor T cells in pathogenesis of common variable hypogammaglobulinaemia. *Lancet*, ii, 609.

WALDMANN, T. A., POLMAR, S. H., BALESTRA, S. T. & others (1972) Immunoglobulin E in immunologic deficiency diseases. II. Serum IgE concentration of patients with acquired hypogammaglobulinemia, chymoma and hypogammaglobulinemia, myotonic dystrophy, intestinal lymphangiectasis and Wiskott-Aldrich syndrome. *J. Immunol.*, **109**, 304.

WU, L. Y., LAWTON, A. R. & COOPER, M. D. (1973) Differentiation capacity of cultured B lymphocytes from immunodeficient patients. *J. Clin. Invest.*, **52**, 3180.